# Statements About the Pervasiveness of Behavior Require Data About the Pervasiveness of Behavior

**DOI:** 10.3389/fpsyg.2020.594675

**Published:** 2020-11-19

**Authors:** Craig P. Speelman, Marek McGann

**Affiliations:** ^1^School of Arts and Humanities, Edith Cowan University, Joondalup, WA, Australia; ^2^Mary Immaculate College, University of Limerick, Limerick, Ireland

**Keywords:** scientific practice, ergodicity, validity, individuality, pervasiveness

## Abstract

Despite recent close attention to issues related to the reliability of psychological research (e.g., the replication crisis), issues of the validity of this research have not been considered to the same extent. This paper highlights an issue that calls into question the validity of the common research practice of studying samples of individuals, and using sample-based statistics to infer generalizations that are applied not only to the parent population, but to individuals. The lack of ergodicity in human data means that such generalizations are not justified. This problem is illustrated with respect to two common scenarios in psychological research that raise questions for the sorts of theories that are typically proposed to explain human behavior and cognition. The paper presents a method of data analysis that requires closer attention to the range of behaviors exhibited by individuals in our research to determine the pervasiveness of effects observed in sample data. Such an approach to data analysis will produce results that are more in tune with the types of generalizations typical in reports of psychological research than mainstream analysis methods.

## Introduction

Much psychological research suffers from a logical problem, which we call the ergodic fallacy. That is, the methods we use to evaluate the outcomes of psychological research examine group-level phenomena (such as means) and do not provide much information on the phenomena of most interest to, for instance, applied psychologists (such as the experiences of individual people) (Rose, 2016; Fisher et al., 2018; Schmiedek et al., 2020). This is not a trivial, but a fundamental problem, and one that has seen increasing recognition amongst methodologists, along with proposed means of overcoming it (Molenaar, 2004, 2007; Grice et al., 2006, 2015, 2017; Molenaar and Campbell, 2009). Despite such recognition, and existence of alternative methods that seem to better suit the kinds of inference in which psychologists are interested, the discussion of this problem has remained relatively niche, and the methods not widely adopted.

In this paper we approach the issue step-by-step. Drawing on the work of others we outline the ergodic fallacy, and its implications for psychological research and practice in a clear and non-technical manner. We offer two detailed examples of empirical studies in the mode of mainstream psychological research to illustrate the problems of ergodic thinking, and the mismatch between the methods that we tend to apply, and the kinds of things in which we are interested in knowing, based on typical phrases and wordings of conclusions in (particularly applied) research papers.

Following that, we introduce *pervasiveness* as a non-technical approach to estimating the presence of an effect within a sample of participants in an experiment. Using a detailed example, we illustrate how such a technique can help to get past ergodic thinking and unlock a wealth of untapped potential in already-existing datasets, in a way that offers valuable insights into psychological phenomena.

We close with a brief description of several benefits that accrue from the use of such a pervasiveness analysis. As a prelude, these advantages include: Pervasiveness analyses provide a superior measure of effect size, with results closer to what we typically want to say about our data; pervasiveness estimates can be easily combined from different studies in a manner akin to performing a meta-analysis; Pervasiveness enables estimates of replicability; and, pervasiveness analyses may indicate the presence of moderator variables.

## Individuals Are Not Groups: Psychology’s Ergodicity Problem

Psychological research is diverse, with various methods deployed to address a range of different kinds of research questions. Perhaps the majority of research in the discipline, though, involves the sampling of groups of people from a defined population in order to learn something about that population. Given the nature of the discipline, however, it is rare that psychological researchers are only interested in answering demographic questions that describe some generality about the population as a whole (e.g., that Y% of the population in country X are not in favor of Easter eggs being displayed for sale in January). More often, we are interested in extrapolating from what we learn about the population, in order to draw conclusions about the individuals who constitute it. Too often we measure collections of individuals in order to theorize about single individuals.

When it comes to studying samples, it is usually a case of “the larger the better.” Given that some error in any particular measure is unavoidable, measuring the same thing many times, and taking the average, is done in order to avoid the bias that might result from over-interpreting a single flawed measurement. The larger the sample, the more likely we are to average out random errors, and the more likely the variable in the sample is to represent the parameter in the population as a whole. A bigger sample makes us more confident both in the accuracy of our measurement, and its generalizability to the population as a whole.

Relatedly, statistical power increases with sample size, such that where our concerns are those of statistical significance, random fluctuations in our sample are less likely to skew the outcomes of a null hypothesis significance test (NHST), improving the reliability of the outcomes of the experiment or quasi-experiment in which we are engaged.

Sample-level statistics, even when dealing with large samples, still come with the costs and assumptions we have detailed elsewhere (Speelman and McGann, 2013). These issues have been discussed to varying extents within our scientific profession for at least the last 60 years (e.g., Cronbach, 1957), and there has been a recent resurgence in interest (Rose, 2016; Fisher et al., 2018). Molenaar (2004, 2007; see also Molenaar and Campbell, 2009), and others (e.g., Hamaker, 2012; Rose, 2016) have fought to raise awareness that, in counter-balance to the apparent benefits, the cost of using samples and averages in order to estimate psychological variables is that almost all comparison and theorizing of those variables must occur at that sample level. Psychological theories in this case must be actuarial descriptions of the behaviors of populations, not specific descriptions or explanations of the behavior of individual people. In other words, mean-based statistics only allow generalizations to the means of other samples, not to individuals. We can draw conclusions about the individual members of those samples only in very specific circumstances, where we can show that the sample in question (as a system of variables) can be considered “ergodic.”

The ergodic theorem, proven by the mathematician Birkhoff, 1931, shows that the behavior of a sample as a whole can be used to predict the behavior of its individual components only in those cases where two criteria apply: (1) all of the members of the sample are essentially interchangeable with one another; and, (2) the members of the sample, and the sample’s average behavior, do not change over time. [More technically, the average of the sample’s behavior over time is the same as the average of all of its potential behaviors at any given moment. For other versions of these two criteria, see Molenaar (2007) and Schmiedek et al. (2020)].

On the basis of these criteria, it is clear that human beings, and groups of them, are quintessential examples of *non-ergodic systems*. No two human beings (even monozygotic twins) are so identical as to be interchangeable on most variables. In addition, people’s tendency to change over time not only over long developmental timescales, but also the shorter timescales of practice and learning, is a perennial thorn in the side of within-subjects designs (Molenaar, 2007). And yet, any method of analysis that involves measuring a sample of people on some dimension and using the average value as a predictor of individual behavior or, crucially, as a description of individual psychological function will be assuming that the system observed is ergodic. In such circumstances, psychologists are committing what Molenaar (as described to Rose, 2016, p. 64) refers to as the “ergodic switch” – studying something that is not ergodic and acting as if it were, and we refer to it here as the ‘ergodic fallacy.’ In a non-ergodic system there is no reliable connection between the results of the sample-level statistics and those of the individuals that comprise those samples. We can generate means from raw data, but we cannot generate those raw data from their means. This fact matters a great deal when we are trying to develop theories that describe, explain, and predict the behavior of individual people from the calculated measurements of many of those people (Hamaker, 2012).

Fisher et al. (2018) have recently provided a clear and substantial illustration of the falsity of ergodic assumptions in six data sets from what would typically be considered high quality research projects in psychology. Re-examining these data sets, they were able to investigate the differences between statistics calculated over samples as a whole, and over the measurements of the individual members of those samples. They found that individual participants were much more variable than the samples they belonged to, with intra-individual variance ranging from two to four times that of samples constituted by those individuals.

Though Fisher et al.’s (2018) study is a very positive sign of growing awareness of this issue, the problem is not a new one, and has been raised repeatedly by others to little apparent effect on mainstream research practices or standard interpretation of findings (Estes, 1960; Lykken, 1991; Danziger, 1994; Grice et al., 2006; Smedslund, 2015; Grice et al., 2017). It is possible that the message has been lost partly in the method of presentation, which has for the main part been couched in fairly dry (if thorough and comprehensive) statistical argument and formulae (e.g., Haaf and Rouder, 2019; Williams et al., 2020). At the least, the solutions provided by others to the ergodicity fallacy have clearly lacked the “cut through” to shift mainstream methods of analysis. But this is not something that we can afford to ignore, confident that our methods and analyses are robust to the violation of this assumption. It speaks to some of the most common forms of psychological research, and as such, the ergodic fallacy is substantially responsible for the breaking of the vital feedback loop between the phenomena proper to the discipline, and the theories purported to explain them.

In the hope of providing a clear illustration of the problems raised by the ergodic fallacy for mainstream scientific practice in psychology, we present two scenarios that demonstrate how the problem manifests in many common research practices. These scenarios not only highlight interpretation issues associated with the ergodic fallacy, but point to a further possible explanation for why awareness of the problem has not translated into changes in research practice. That is, researchers may not have known what to do to address the problem.

Properly responding to this challenge will require a number of concerted actions, but these need not all be heavily technical, and all will help us grasp the relationship between phenomena and data more clearly.

## Scenario 1: What Can an Experiment Tell Us About Real-World Behavior?

Imagine we were trying to discover a method for studying new learning material that would improve both comprehension and retention. Let’s say that we wanted to compare a new study technique with a more traditional method. The standard approach to exploring this issue would be to conduct an experiment. Participants in the experiment would be recruited, typically from a university undergraduate population, on the assumption that university students are fairly representative of the general population and so whatever we observe with them should generalize well.

The participants would then be randomly allocated to one of the two experimental conditions. Both groups would be exposed to some material that they are unlikely to have experienced before, let’s say a recorded lecture on the history of Mesopotamia. Afterward, the groups could be given a quiz on the material. After the quiz, one group would be given a transcript of the lecture and asked to study the material with a traditional method, such as reading and re-reading it, for 30 min. The second group would also be given the transcript but would be instructed to study the material according to the new technique, also for 30 min. At the end of the study period, both groups would then be given the quiz again.

To determine whether the two study techniques had different effects on the ability to perform on the quiz we could calculate a difference score for each person, comparing their result on the first quiz to their result on the second quiz. Typically, the difference scores for the two groups would be compared with some form of NHST, such as a *t*-test. Other analysis methods could also be used, such as a mixed design ANOVA with study technique as a between-subjects variable, and quiz (first vs. second) as a within-subjects variable. In this case, an interaction between the two variables would indicate that the two techniques differed in their effect on learning (The relevant *F*-value and the aforementioned *t*-value are arithmetically related: *t*^2^ = *F*). Of course, there are Bayesian alternatives to these sorts of analysis (e.g., Nathoo and Masson, 2016), and many other techniques that would examine the data in a myriad of ways. They would all share the one fundamental aim, however, and that is to try to detect whether or not performance in the two conditions was different.

Imagine that the statistical analyses of these data all indicated that the new study technique was associated with a greater improvement on quiz performance than the traditional technique. How would psychologists typically report such a result in a journal paper or a conference presentation? Following presentation of all of the statistical information and a general summary of the results at the beginning of the Discussion, we would expect to see or hear something like the following: “*The new study technique led to better learning than the traditional study technique.*”

Usually when the conclusion has been stated, there is scope for some discussion of what the result means, practically and/or theoretically. In this case the researcher could expound on how the new study technique could be adopted in the classroom to help students learn more effectively. The researcher might also feel confident about commenting on the status of some theory about how memory works, and that the new study technique supports a central claim of the theory because it exploited a cognitive mechanism that is described by the theory. It is in this sort of discussion where the major problems for sample-based experimental psychology emerge. The fashion is not only to extrapolate the results of an experiment to a wider population – the result found in the sample should also be found in the population – but also to the individuals in that population. That is, the effect observed in the experiment should be observable in each individual. In this case that would mean that every person in the population would be better off studying with the new technique than with the traditional technique. Underlying these conclusions is an implicit logic, un-tested by the experiment in question, that each person has the same cognitive mechanism operating in their memory that the new study technique exploits.

Initially the reader might claim that we have gone too far with this characterization of experimental protocol; that no one claims that experimental results apply to everyone in a population. This is true, but we would argue that what is equally true is that almost no one makes claims, or offers any analysis, about the extent to which generalizations can apply to the individuals within the population. That is, what proportion of the population can be described by the results of any particular experiment?

In the scenario just described, within the sample of those students who used the new study technique, it is quite possible that some participants’ performance was unaffected, or even became worse. This complexity and texture in the behavior of individuals is suppressed by the use of tests of mean difference, and by typical results reporting procedures that provide little to no information regarding this texture and variability in the data.

With respect to this hypothetical experiment, it is valid to say that if we conducted another study on a similar sample from the same population, we could expect to see the same sort of overall effect (notwithstanding the replication issues that have been much publicized recently – Ferguson, 2015 – as well as the statistical limits on replication identified by Cumming, 2008). It is not, however, valid to say that if someone wanted to study some new material, they would be better off utilizing the new study technique. Because collections of human beings are not ergodic, we have very little evidence about individual behavior at all.

For example, imagine an educational psychologist read an account of our experiment with the conclusions presented above. She might interpret the results as advice she could apply to students at her school. Imagine that a teacher at the school has identified a few students who appear to have difficulties learning the material he is covering in his class. On the basis of the conclusions from our experiment, the psychologist decides to advise the students about the new study technique. What are the chances that this will be a successful strategy, and the students’ academic performance in that class will improve? Even if their initial problems with studying were based solely on the inadequacy of the traditional study technique (i.e., we are ignoring possible social, economic, behavioral, cognitive etc. issues), there is nothing in the results of the experiment that would enable us to estimate the probability that adopting the new study technique would result in improved performance. The best that we could hope for is that the overall performance of this new sample might improve, but we should not be confident that every person in the sample will improve. The reason is that the experiment only provided information about samples.

Maybe if the norm in psychological research was to report individual data, such as the number of people in one condition who out-performed members of the other condition, the educational psychologist could estimate the likelihood that the students she was dealing with would improve. But even that would, no doubt, fall short of her expectations of applying a new study technique. Surely her aim would be to help all of the students, not just to increase the average performance of the group. Judging by the modally occurring forms of research practice in experimental research, it would seem that psychology has settled for the latter, even though we profess to being able to provide direction on the former. One of the outcomes of this fact is the strained relationship, and often cynicism, that exists in applied domains of practice, such as education, for the outcomes of academic research, which are seen as generally neither conclusive, nor practical (Broekkamp and van Hout-Wolters, 2007; IJzerman et al., 2020). This may reflect insufficient attention to the existence of moderating variables that can define subgroups who may respond differently. The method of analysis we outline below has the potential to simplify searching for such variables.

The failure of the ergodicity assumption not only means that analyses based on measurements of samples alone provide very little information about individuals, it forces us to recognize, and call into question, a further assumption underlying much of contemporary research in cognitive psychology – that we all have the same cognitive system.

It is common in cognitive psychology to propose mechanistic models of cognitive processes as explanations for why experimental conditions resulted in particular types of performance. Unfortunately, testing such models on the average performance of samples is only testing whether the sample possesses such a set of processes. The modelers would presumably like to suggest that their models provide an explanation for the performance of each individual in the experiment, as if each individual has a set of cognitive processes as described by the model (Grice et al., 2017). As indicated above, researchers rarely report the extent to which individual performance is consistent with sample-level effects, and so it is not possible to evaluate whether such models are good descriptions of individual cognition.

Certainly, if researchers did report such information, it would be clear that not everyone in a sample exhibits the same performance pattern as the sample, and indeed some may even exhibit the opposite pattern (e.g., Molenaar, 2004; Molenaar and Campbell, 2009; Speelman and McGann, 2013; Grice et al., 2017), a result that would count against a general model of cognition. We suspect that such an outcome would be written off as ‘experimental error,’ and the typical justification for use of a large sample is precisely to reduce error. But such an attitude reflects the assumption that everyone shares the same cognitive apparatus, and our experiments are designed to examine this through the noise that is inherent in human data. The methods we use to undertake this examination are not only based on this assumption, but they are designed to reinforce it. As a result, these methods are not conducive to exploring an alternative state of affairs, such as, for instance, the possibility that humans develop idiosyncratic cognitive processes as a result of adapting to their world (Lykken, 1991; Van Orden et al., 2003; Speelman and Kirsner, 2005; Molenaar, 2007). This limitation of our analysis methods is illustrated in the next scenario.

## Scenario 2: How Pervasive Is a Significant Effect?

Let us consider in greater detail the situation where a sample-level effect is not revealed in the performance of all of the individual members of the sample. To explore this situation, we generated some hypothetical data to simulate a scenario where a sample of 50 people performed a test on two occasions. Imagine that we are interested in whether the scores increase from the first test to the second test (i.e., the scores improve). To simulate such a scenario, we used Microsoft Excel to generate 50 random numbers between 1 and 100 to act as difference scores (i.e., test 2 – test 1). These difference scores are presented in Table 1 as Set A.

**TABLE 1:** Data sets discussed in Scenario 2.

Data																			
Set A					Set B					Set C					Set D				
57	78	81	57	6	37	78	81	57	26	17	78	81	57	46	−3	78	81	57	66
77	89	53	52	70	57	89	53	52	90	37	89	53	52	110	17	89	53	52	130
31	43	61	25	37	11	43	61	25	57	−9	43	61	25	77	−29	43	61	25	97
42	70	98	10	80	22	70	98	10	100	2	70	98	10	120	−18	70	98	10	140
76	16	33	65	100	56	16	33	65	120	36	16	33	65	140	16	16	33	65	160
39	98	62	24	99	19	98	62	24	119	−1	98	62	24	139	−21	98	62	24	159
81	59	54	91	33	61	59	54	91	53	41	59	54	91	73	21	59	54	91	93
45	46	28	49	1	25	46	28	49	21	5	46	28	49	41	−15	46	28	49	61
77	76	40	78	79	57	76	40	78	99	37	76	40	78	119	17	76	40	78	139
23	44	81	55	99	3	44	81	55	119	−17	44	81	55	139	−37	44	81	55	159
Mean		57.36					57.36					57.36					57.36		
*SD*		26.36					30.07					37.94					47.97		
*t*		15.38					13.49					10.69					8.45		
*p*		<0.001					<0.001					<0.001					<0.001		

**Data Set A is 50 numbers generated randomly with the constraint that the range be 1–100. Data Set B is the same Data Set A except the first column of numbers are smaller by 20, and the fifth column of numbers are larger by 20. The same pattern of difference exists between Data Sets B and C, and between Data Sets C and D. All data sets are normal according to Kolmogorov–Smirnov (p > 0.05) and Shapiro–Wilk (p > 0.05) statistics*.*

The fact that all numbers in Set A are positive indicate that all participants improved on test 2 compared to their performance on test 1. Clearly, some participants improved to a greater extent than others. Straightforwardly though, as indicated by a *t-*test that was performed to test the null hypothesis that there was no improvement, a significant improvement was detected.

**FIGURE 1:**
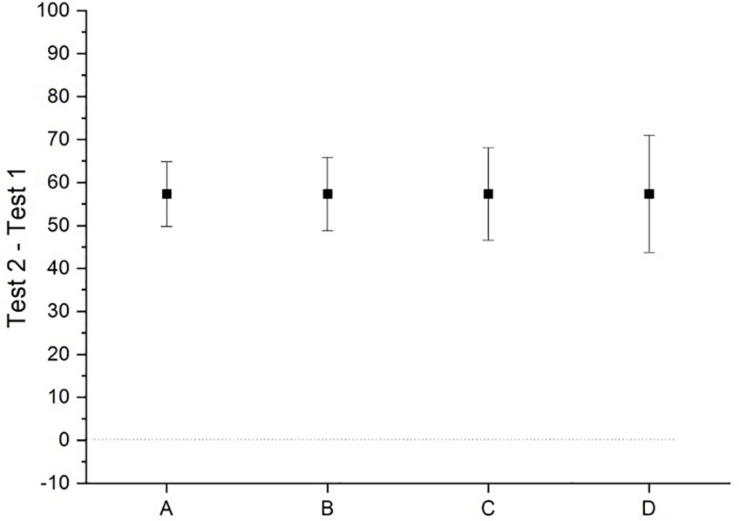
A graph of the mean values and their 95% confidence intervals for the hypothetical data presented in Table 1.

**FIGURE 2:**
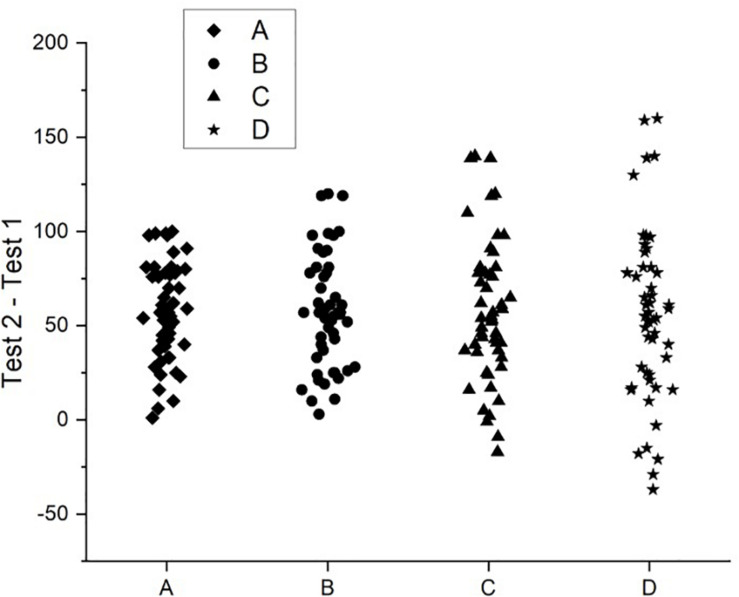
A graph of the hypothetical data presented in Table 1.

Consider now the other data sets presented in Table 1. Each one is derived from Set A. For instance, the scores of the first 10 people in B are 20 less than the first 10 in A. The next 30 people (i.e., columns 2–4) in B have the same scores as the people in these columns in A. The final 10 people in B are 20 more than the final 10 in A. Sets C and D have been derived similarly, but the differences are 40 and 60, respectively. Sets B, C, and D were derived in this way to ensure that the mean improvement scores were the same as in A. That is, the average improvement in all sets was the same. Indeed, *t*-tests for each set returned statistically significant results, indicating that there was a significant improvement in each set. Furthermore, 95% confidence intervals around these mean improvement values are a long way from overlapping with the value that would indicate no improvement (i.e., zero) (see Figure 1). And yet, careful inspection of each set indicates that improvement is not uniform across the sets (see Figure 2). Where there is ubiquitous improvement in Sets A and B, in Set C some people show a worsening of performance, and others show an extreme amount of improvement. This pattern is magnified in Set D.

The point of this scenario is to demonstrate that, when we use NHST or similar tests to analyze our data at the sample level, an effect that is very large in extent in some individuals can outweigh the null or opposite effects in others, and this can result in an overall effect at the sample level (without this simply being a result of one or two notable outliers). That is, a large effect may exist in some individuals, but others can demonstrate quite a different effect or no effect. Nonetheless, we venture to suggest that many researchers would conclude similar things about each data set and its associated analysis if they had any one of the sets as the outcome of their own experiment, and that is that they have detected a general effect of the IV (i.e., test 1 vs. test 2). All of these data sets might return similar outcomes from significance testing (i.e., reject the null hypothesis), but which one leads to greater confidence that the effect exists? Sets A and B surely would give us more confidence that we have observed a *universal* effect. Sets C and D suggest the effect may have caveats (i.e., it only exists in certain people).

As researchers, we are not usually in the position of being able to compare experimental outcomes such as those presented in this scenario. Instead, we only have one sample to explore, and so we usually base our judgments about an effect on some common indicators about the sample. For instance, we might explore the data to ensure that the distribution of scores is normal (it is in all of the sets presented here – see information provided in Table 1), and once satisfied, continue on to an inferential statistics test of some sort. When the test suggests rejecting the null hypothesis, we would probably be satisfied that a test effect had been observed. We might, however, be interested in the size of the effect.

Although the size of the effects in Sets A to D vary because of the different variances in each sample, they all exceed 1 standard deviation, which is usually considered a large effect. So, many researchers would likely conclude the same thing regardless of which data set they had at their disposal, which is, that an overall effect was observed. The common approach to analysis of relying on aggregating statistics can result in situations such as those in Sets C and D being overlooked. At the very least, our standard reporting techniques and modes of discussion of results suppress a reader’s ability to make these judgments for themselves, and are dependent on the researchers noticing them and explicitly choosing to include them in their presentation of the experiments.

In response to the example provided above, one reviewer of an earlier version of this manuscript claimed that we seem to “ignore the fact that statistical tests are often accompanied by effect size measures, which reflect how much overlap exists between groups.” As should be obvious from the example, we are fully aware that effect size measures are traditionally reported, and that they provide some information about overlap between groups, but this is our point exactly. Such measures only reveal information about group overlap, and nothing about overlap between individuals within groups. Effect size measures are summary statistics just like the mean and variance, and so reflect group characteristics and can be largely insensitive to dramatic differences amongst individuals as seen in Sets C and D above. Presenting information about individual performance in a figure such as Figure 2 is far more effective in conveying overlap between individuals within groups than standard effect size measures. Similar arguments were made many years ago by Tukey (1977).

To many readers these points are nothing new. We have already noted that psychologists are carefully examining the limitations of our research methods and statistics (Gigerenzer, 2004; Simmons et al., 2011; Lakens, 2019). More specifically, the issues concerning ergodicity and conflict between individual and group levels of analysis has a substantive existing literature as we have already noted (Molenaar, 2004, 2007; Molenaar and Campbell, 2009; Hamaker, 2012; Rose, 2016; Fisher et al., 2018; Logie, 2018). What is perhaps most concerning to us is that this literature has had very little impact. Some researchers may well be aware of the issue, but feel it is not a problem because it is well-accepted that sample-level effects are not demonstrated in every individual, but are demonstrated in most people. In this case, we should ask how do they know this? Establishing that most people in a sample behave the same way is rarely reported in journal papers – descriptive statistics rarely include this level of detail (though it would be simple to do so, and the analysis method we present below provides one way for doing this). Certainly, there is no clear definition of ‘most.’

It would appear that researchers in sample-based experimental psychology are often unaware of this conundrum. It is common to read conclusions in reports of psychological experiments like the following: “…people integrate ensemble information about the group average expression when they make judgments of individual faces’ expressions” (Griffiths et al., 2018, p. 311, Abstract). In this particular instance, it is not clearly stated what is meant by ‘people’? Does this mean ‘all people’? Or are the authors more realistic, and mean ‘most people’? But again, how can researchers be confident that they have observed something that exists in the majority? And more specifically, does this mean 90%, or 80%, or just over 50%? (Note that these authors are cited purely as an illustration – a recent issue from any empirical psychology journal will likely provide others. Textbooks also provide countless examples of generalizing research outcomes to ‘people’).

When there has been some statistically significant effect observed, it is not the fashion to report the number of people in the study that showed the effect. So, researchers are generalizing from a sample-level effect to ‘people.’ It is problematically unclear as to precisely how such claims should be parsed, and if it is intended to imply numerous ‘individual people,’ then it is simply not supported by the kinds of evidence typical to published research reports. With respect to the data depicted in Figures 1, 2, Set A would justify a statement such as ‘people improve under these circumstances,’ whereas Set D would not justify such a statement.

Some authors have recently tackled the relationship between individual level and sample level effects. To this end, for example, Miller and Schwarz (2018) have proposed interrogating null results in ANOVA designs more closely to determine whether the null effect exists in all participants, or whether some people in the sample show an effect. This approach has also been advocated by Smith and Little (2018). Miller and Schwarz describe a new method for examining interactions in ANOVAs to achieve this goal, whereas Smith and Little propose a small-n approach in which the number of people showing an effect is an explicit object of investigation. Although both approaches are suggestions in what we think is the right direction, these authors focused on null results. But what of non-null results, which are the majority of those published (Nosek et al., 2012; Ioannidis et al., 2014; Ferguson, 2015; Smaldino and McElreath, 2016)? We reiterate that it is just as important to examine carefully the pattern of individual effects underlying any sample effect.

## Individual Differences Research

A predictable response to the issues we have highlighted here is to say that there is a long tradition in Psychology of examining individual differences (Cronbach, 1957; Underwood, 1975). Certainly a great deal of research has been reported regarding the effects on behavior and performance of variables such as intelligence (e.g., Côté and Miners, 2006), personality (e.g., Neuman and Wright, 1999), working memory capacity (WMC) (e.g., Van Merriënboer and Sweller, 2005) and age (e.g., West et al., 1992). In the main, such research involves examining the effects of such variables as correlations with some measure of behavior, leading to such statements as “the greater someone’s WMC, the greater their capacity for performing mental arithmetic.” But is this really examining individual differences in a way that would uncover whether individuals within a group are different from each other, and the nature of such differences?

Consider the type of data that are collected to perform such a correlational analysis. To examine the WMC/mental arithmetic relationship, for instance, a researcher need only obtain a measure of WMC and a measure of mental arithmetic performance from each person in a sample. Thus each person would contribute a pair of numbers to the calculation of the correlation estimate. On the basis of the whole sample of such pairs, the extent to which the two variables are related could be determined. Thus, some sort of relationship between the variables might be discovered (if one exists), such as large values in one variable are associated with large values in the other variable. Typically this would suggest that a systematic relationship between the two variables has been discovered, which could be used to characterize the original data set, or possibly to make predictions about the performance of other samples. A correlation coefficient may be used to characterize this relationship, however, a scatterplot provides a far more informative representation of the relationship across all measured values of the variables.

Researchers who engage in individual differences studies like those we have just described clearly have some appreciation of the fact that there is often large individual variation within samples. It is important to note, though, that this approach is still a summative one, where the relationship between variables is used to characterize the whole sample. It is possible to use such relationships to separate groups on the basis of their performance (e.g., in a positive correlation between variables A and B, one can identify those who score high on A are likely to also score high on B), but such information falls short of being able to make predictions about how likely it is that someone will obtain a particular score on one of the variables (Cronbach, 1975; Lamiell, 1997, 2013).

## Toward Improved Practice

While we raise grave concerns about some common (though certainly not universal) practices of sample-based experimental psychology here, we do not argue that sample means should be of no interest to psychology, nor that research to date does not offer us important insights. Indeed, it is quite possible that all of the raw data that have been collected to date have more to offer our research community than has been reported thus far. There are details of individual variability, frequencies of individuals showing changes or effects, and other details that can provide us with a much more rounded description of the phenomena in which we are interested. But it is important to realize that averages describe systems different to the individuals that make up those systems. In order to understand them, we will not only have to study the individuals as individuals more frequently, but will also need to develop a set of theoretical resources (currently unavailable for most domains, such as cognitive psychology) for understanding the relationship between the individuals and the samples in which they participate. In doing so, it will greatly improve the coherence between psychological phenomena as they occur in controlled settings, and as they occur “in the wild” (Hutchins, 1995; see also Cialdini, 2009; McGann and Speelman, 2020).

Some openly admit that psychology can never provide clear predictions about individual behavior because it is a science that enables actuarial predictions rather than clinical ones (Lykken, 1991; Stanovich, 2013). The best that can be provided is some estimate of the probability of a person behaving in a certain manner under certain circumstances. For example, a person has a 70% chance of exhibiting behavior *X* when they are faced with situation *Y*. Stanovich states that “virtually all of the facts and relationships that have been uncovered by the science of psychology are stated in terms of probabilities” (p. 155). If this is true, then it might be a useful lesson that could drive change in the types of misleading prescriptions psychologists and other human scientists are prone to in reporting conclusions from their research (Boutron, 2020; Reynolds-Vaughn et al., 2020). We, however, take issue with this statement. It is not the case that most psychological research is stated in terms of probabilities that would enable a statistical prediction of a behavioral outcome.

Such predictions can be derived from sample-level results of research, but only if the results have been analyzed and reported in a particular way. If the research involves a comparison between samples in terms of some measure on a scale, and the conclusions are based on a statistically significant difference between the samples on that measure, then such a result can tell us nothing about the likelihood of someone exhibiting a specific behavior. A *p*-value that follows from an NHST, for instance, is in no way connected with the probability associated with an actuarial prediction. What is required in order to make such actuarial predictions is for the original research to have determined how many people in the sample exhibited the specific behavior under one set of conditions compared to the number that demonstrated that behavior under another (control) set of conditions. A great deal of sample-based experimental psychology research fails to consider this question. Researchers seem to assume that all that is necessary to enable the actuarial predictions mentioned above is to demonstrate a significant difference on some measure of behavior. On the contrary, what is required is, first, some defined point on that measure that represents the “target” behavior, and second, a simple count of the number of people in the various experimental groups that exhibit this behavior, and those that do not.

So, if actuarial prediction is the highest ambition for the science of sample-based experimental psychology (and it may well be that actuaries, or perhaps meteorologists should be used as models of good practice for psychology), at present the field falls far short of even this mark. This is because the basic nature of sample-based experimental psychology research is aimed at producing sample-based generalizations that cannot be construed as probabilistic outcomes. A different approach to the analysis of experimental psychology data is required in order to produce results that may enable more principled predictions.

It turns out that there may be a great deal to be gained from focussing more on intensive examination of individual behavior. This was *de rigeur* in the early history of psychology (Danziger, 1994; Gigerenzer, 1998), and has been a regular feature of research into perceptual processes (Smith and Little, 2018). There will still be a need for studying collections of individuals, but there must be a close focus on the data produced by each individual so as to determine the pervasiveness of particular behavior patterns, and the possibility that differences in behavior within a sample are more likely to be seen in particular sub-samples identifiable by some shared characteristic. Such data will better inform generalization statements, and possible applications beyond the research field. And large data samples will still be important. For instance, we venture to suggest that a conclusion that 80% of people demonstrate a particular effect will be far more convincing when it is derived from a sample of 500 people than if it were derived from a sample of 10 people.

## Analysis of Pervasiveness

As indicated earlier, we suggest that one of the reasons why researchers who were aware of the ergodic problem may have been reluctant to move away from traditional sample-based experimental psychology designs is that they were unaware of any easily applied or understood alternative methods. Below we describe one method for addressing the ergodic problem in a sub-set of experimental psychology designs that researchers may find relatively straightforward to apply. The design used in the example is simple, but it reveals the required evidence for common conclusions. More complicated designs can be analyzed to provide such evidence, but more sophisticated techniques are required [e.g., Observation Oriented Modeling (OOM), Grice, 2011, 2015; Grice et al., 2015]. The method for analyzing the pervasiveness of an effect we demonstrate below can be considered a specific case of an Ordinal Pattern Analysis (OPA) (Thorngate and Carroll, 1986; Craig and Abramson, 2018). Thus, this method is not a new one, however, we present it in a simple manner to demonstrate both the need for such an approach to data analysis, and the sorts of conclusions such an analysis can enable.

For a method of data analysis to be useful it should reflect the types of conclusions that researchers generally report, but often do not have the evidence to support (see Thorngate and Carroll, 1986; Molenaar, 2007). For example, “people behave in *X* fashion under *Y* circumstances” – such a statement carries with it the implication that *most* people behave in *X* fashion, however, researchers rarely substantiate such an implication with relevant evidence. What is required to support such statements is some indication of how many people were observed to behave in this way (Sauer, 2018). Equally useful would be some indication of how many people did not behave this way. This can be reported directly, or the number of people behaving in the target manner can be reported as a proportion of the total number of people observed. There may also be good reason to report the number of people who behaved in the opposite manner (if that makes sense in the context). Above all, this method of analysis should be a measure of the pervasiveness of the target behavior.

To achieve the aims of the pervasiveness analysis, we need at least three things:

### Precise Definitions of Behavior

In practice, this will require more than just a clear description of the behavior. It will often require that a precise criterion is defined whereby we can determine if the behavior has been observed, or not. For instance, we may be interested in whether or not a person has exhibited a particular effect (e.g., the Stroop Effect). To achieve the aims we have specified for the pervasiveness measure, it will be necessary to be able to decide from an individual’s performance data whether that individual exhibited the effect. Traditionally psychologists make this judgment on the basis of a sample of people, and make decisions about whether or not an effect was observed *in the sample*. The pervasiveness measure requires that this can be done with each individual. How this is done requires some significant thinking about what constitutes an effect, and this should include how large an effect is before we conclude that an effect has been observed. We fully acknowledge that this could be a difficult task, but we argue that such considerations will ultimately be valuable in increasing the confidence in the validity and reliability of our research results. We illustrate the types of necessary considerations below in an example.

### Count the Number of People

When we have a clear definition of what constitutes the target behavior, it is a simple matter then to count the number of people in our sample that exhibited this behavior. Most of the time this will imply the number of people who did not show the behavior, although sometimes it might be useful to introduce greater granularity in the counting. For example, a researcher might be interested in the number of people that exhibit a particular effect (e.g., performance on *X* in condition *A* is better than in condition *B*: *X*_*A*_ > *X*_*B*_) and distinguish this number from the number that show no such effect (*X*_*A*_ = *X*_*B*_) and those that show the opposite effect (*X*_*A*_ < *X*_*B*_). Further, there could also be occasions where the behavior has a variety of definitions that vary in extent (e.g., the effect is observed when the differences in performance exceed 10% vs. the effect is observed when the differences in performance exceed 20%), and so the number of people that fall into the relevant categories under the respective conditions will be required.

### Define ‘Majority’

When we have determined the number of people in our sample that have exhibited an effect, we should then be interested in how best to characterize that number. For instance, does this number represent the ‘majority’ of people in the sample? To answer this question requires a definition of ‘majority,’ a pre-defined proportion value corresponding to our idea of “most people.” In our view, this is a value that would convince others that what we have observed is “pervasive,” and presumably, worthy of theoretical and practical interest. For instance, “80% of people showing the effect” might be considered convincing evidence that “the effect exists in most people.” Before we are criticized for attempting to replace one strict criterion (α = 0.05) with another strict criterion, this 80% value could be considered a benchmark value that signals a degree of confidence that we have observed a pervasive phenomenon. That is, if the proportion of a sample that behaves in a particular manner is 80% or more, it is unlikely that many people will argue if this result is characterized as “most participants behaved in this manner.” As the proportion falls below this value, however, more people are likely to take issue if the result is described as a characteristic of ‘most’ of the sample. Thus the pervasiveness value can be viewed as the degree to which an assertion about the existence of an effect is likely to be considered convincing by others. This value is likely to change dependent on a range of factors, including the controversial nature of the claim.

## Analysis of Pervasiveness: A Worked Example

Some years ago, one of us published a report of an experiment designed to test several methods for improving peoples’ skill at determining whether or not a skin lesion is dangerous (Speelman et al., 2010). Details of the methodology and results can be found in that paper, but in essence there were 5 experimental conditions to which 100 participants were allocated randomly (*n* = 20). Two of these conditions involved practice at making decisions about skin lesions. The indicator of whether a condition (the *condition* variable) was effective in improving skill was the difference between performance on a Pre-test and a Post-test (the *test* variable) of making a dangerous/non-dangerous decision about a set of pictures of skin lesions. The conditions were compared in their effect on this skill by conducting ANOVAs on the accuracy (*Acc*) of, and time to make (*RT*), decisions about the lesions. In both analyses, interactions between *condition* and *test* were statistically significant, indicating that the amount of improvement observed from the Pre-test to the Post-test was related to the condition experienced in between the tests.

It is pertinent to focus on the way in which the results of this experiment were characterized in the conclusions. As reported in the paper, “The current experiment found that 30 min of training with several hundred pictures of skin lesions can improve the ability to discriminate between dangerous and non-dangerous skin lesions by 12–15%” (Speelman et al., 2010, p. 293). An obvious question that should be asked of this conclusion is, what is the pervasiveness of this finding? Did everyone in the conditions that showed this overall level of improvement demonstrate a similar amount of improvement? And what of people in the other conditions – did they uniformly show little improvement? As was convention at the time, and, as we argue here, still the case now, no information that could be used to answer these questions was reported in the paper. This is where the pervasiveness analysis can fill the gap between the data we often routinely collect and the conclusions we usually want to make about our experiments.

The mean data values that were analyzed with ANOVA in the original paper are presented in Tables 2, 3. In Table 2, the Pre-test RT is compared with the Post-test RT. Positive RT difference values represent an improvement in the speed of making decisions about the skin lesions. In Table 3, a similar comparison is made between the Pre-test and Post-test Accuracy values, with positive values indicating improved decision accuracy.

**TABLE 2:** RT (*ms*) data from Speelman et al. (2010).

Participant	condition	premean	postmean	diff (pre-post)	Participant	condition	premean	postmean	diff (pre-post)	Participant	condition	premean	postmean	diff (pre-post)
1	1	2013.06	832.26	1180.8	41	3	1368.06	907.16	460.9	81	5	2467.06	1695.74	771.32
2	1	2604.94	1317.04	1287.9	42	3	1904.03	741.24	1162.79	82	5	1996.35	1653.86	342.49
3	1	2648.43	2027.32	621.11	43	3	1339.48	951.54	387.94	83	5	874.71	931.93	–57.22
4	1	2345.89	1038.43	1307.46	44	3	1876.62	981.83	894.79	84	5	1286.4	920.17	366.23
5	1	2241.44	1035.26	1206.18	45	3	1680.52	1171.45	509.07	85	5	1742.49	1425.27	317.22
6	1	2435.8	784.57	1651.23	46	3	2070.37	1692.01	378.36	86	5	2006.94	2074.1	–67.16
7	1	2347.35	772.15	1575.2	47	3	1551.54	800.8	750.74	87	5	3348.83	3361.56	–12.73
8	1	1976.36	718.17	1258.19	48	3	2151.11	1225.61	925.5	88	5	1565.82	1600.44	–34.62
9	1	2591.59	1593.74	997.85	49	3	1932.1	1543.13	388.97	89	5	1936.61	1490.12	446.49
10	1	2213.02	1143.95	1069.07	50	3	3261.32	1218.78	2042.54	90	5	1699.1	1282.89	416.21
11	1	2315.94	924.17	1391.77	51	3	2446.97	1066.47	1380.5	91	5	840.17	771.62	68.55
12	1	2389.63	1291.25	1098.38	52	3	1977.76	926.58	1051.18	92	5	1621.41	1532.9	88.51
13	1	2007.84	1382.1	625.74	53	3	3109.07	1213.93	1895.14	93	5	2083.82	1881.5	202.32
14	1	2091.5	984.35	1107.15	54	3	1866.5	1362.81	503.69	94	5	1508.56	1684.86	–176.3
15	1	2729.48	1086.35	1643.13	55	3	2274.39	1658.72	615.67	95	5	1561.06	1410.25	150.81
16	1	1849.05	1237.83	611.22	56	3	2265.82	1204.99	1060.83	96	5	1676.82	2146.16	–469.34
17	1	2369.29	950.32	1418.97	57	3	1783.42	925.24	858.18	97	5	3001.33	1447.63	1553.7
18	1	2724.26	1199.57	1524.69	58	3	3534.62	1171.8	2362.82	98	5	1790.08	1275.83	514.25
19	1	2555.23	1066.91	1488.32	59	3	2428.83	1164	1264.83	99	5	1929.88	1454.15	475.73
20	1	2512.35	1356.28	1156.07	60	3	1237.35	714.32	523.03	100	5	1145.2	1196.51	–51.31
21	2	3140.15	1802.76	1337.39	61	4	2099.92	1770.96	328.96					
22	2	1645.91	2578.85	–932.94	62	4	2344.75	2166.57	178.18					
23	2	2155.22	1562.05	593.17	63	4	2842.33	2853.46	–11.13					
24	2	2483.86	1971.13	512.73	64	4	1185.57	1107.5	78.07					
25	2	1450.26	1975.1	–524.84	65	4	1861.44	2099.17	–237.73					
26	2	3029.75	2122.21	907.54	66	4	2207.76	1413.79	793.97					
27	2	2261.57	2228.38	33.19	67	4	2679.25	1499.89	1179.36					
28	2	2251.91	1712.87	539.04	68	4	3089.42	1559.58	1529.84					
29	2	1804.35	1681.31	123.04	69	4	2415.24	1629.1	786.14					
30	2	2364.68	2021.7	342.98	70	4	1581.82	931.49	650.33					
31	2	2245.64	2016.78	228.86	71	4	2708.03	2694.96	13.07					
32	2	1778.43	1686.13	92.3	72	4	1017.21	1232.96	–215.75					
33	2	2783.01	1764.77	1018.24	73	4	1737.45	1510.94	226.51					
34	2	2165.08	1964.08	201	74	4	1780.16	1888.63	–108.47					
35	2	1897.53	2097.56	–200.03	75	4	1609.85	1554.2	55.65					
36	2	2084.23	2134.07	–49.84	76	4	1869.37	1193.85	675.52					
37	2	1906.34	1914.46	–8.12	77	4	1419.11	867.9	551.21					
38	2	2017.65	1693.88	323.77	78	4	2139.53	2506.74	–367.21					
39	2	2079.25	2017.6	61.65	79	4	1185.85	1627.96	–442.11					
40	2	2017.91	2016.63	1.28	80	4	2857.52	1498.24	1359.28					

**TABLE 3:** Accuracy (*%*) data from Speelman et al. (2010).

Participant	group	premean	postmean	diff (post - pre)	Participant	group	premean	postmean	diff (post - pre)	Participant	group	premean	postmean	diff (post - pre)
1	1	65.18	80	14.82	41	3	70	73.33	3.33	81	5	63.33	56.67	−6.66
2	1	76.67	73.33	−3.34	42	3	86.67	86.67	0	82	5	76.67	83.33	6.66
3	1	70	64.81	−5.19	43	3	76.67	86.67	10	83	5	83.33	83.33	0
4	1	82.22	70	−12.22	44	3	70	86.67	16.67	84	5	70	67.78	−2.22
5	1	79.26	73.33	−5.93	45	3	83.33	90	6.67	85	5	83.33	76.67	−6.66
6	1	63.33	93.33	30	46	3	76.67	70	−6.67	86	5	56.67	61.11	4.44
7	1	76.67	86.67	10	47	3	80	66.67	−13.33	87	5	70	80	10
8	1	65.18	80	14.82	48	3	86.67	96.67	10	88	5	73.33	75.56	2.23
9	1	72.22	75.55	3.33	49	3	56.67	82.22	25.55	89	5	73.33	73.33	0
10	1	75.18	83.33	8.15	50	3	76.67	80	3.33	90	5	66.67	60	−6.67
11	1	76.67	86.67	10	51	3	60	86.67	26.67	91	5	66.67	76.67	10
12	1	68.52	83.33	14.81	52	3	72.59	80	7.41	92	5	73.33	78.89	5.56
13	1	80	90	10	53	3	55.56	83.33	27.77	93	5	86.67	83.33	−3.34
14	1	54.81	66.67	11.86	54	3	83.33	93.33	10	94	5	90	80	−10
15	1	53.33	76.67	23.34	55	3	58.33	86.67	28.34	95	5	53.33	86.67	33.34
16	1	66.67	86.67	20	56	3	63.33	83.33	20	96	5	73.33	73.33	0
17	1	66.67	83.33	16.66	57	3	82.59	90.61	8.02	97	5	65	70	5
18	1	68.52	90	21.48	58	3	46.67	85.93	39.26	98	5	70	76.67	6.67
19	1	72.59	93.33	20.74	59	3	53.33	85.93	32.6	99	5	89.63	93.33	3.7
20	1	73.33	86.67	13.34	60	3	33.33	86.67	53.34	100	5	58.89	76.67	17.78
21	2	67.59	66.67	−0.92	61	4	82.22	79.63	−2.59					
22	2	63.33	83.33	20	62	4	43.33	60	16.67					
23	2	72.22	60	−12.22	63	4	53.33	69.26	15.93					
24	2	68.15	46.67	−21.48	64	4	76.67	86.67	10					
25	2	61.85	73.33	11.48	65	4	63.33	56.67	−6.66					
26	2	71.48	66.67	−4.81	66	4	66.67	75.41	8.74					
27	2	72.22	63.33	−8.89	67	4	68.15	65.55	−2.6					
28	2	72.96	66.67	−6.29	68	4	43.33	55.18	11.85					
29	2	75.92	86.67	10.75	69	4	66.67	83.33	16.66					
30	2	78.52	70	−8.52	70	4	69.26	63.33	−5.93					
31	2	90	66.67	−23.33	71	4	73.33	82.96	9.63					
32	2	86.67	80	−6.67	72	4	80	83.33	3.33					
33	2	54.44	90	35.56	73	4	86.67	53.33	−33.34					
34	2	82.96	63.33	−19.63	74	4	90	83.33	−6.67					
35	2	86.67	76.67	−10	75	4	69.26	70	0.74					
36	2	76.67	66.67	−10	76	4	20	96.67	76.67					
37	2	83.33	83.33	0	77	4	60	70	10					
38	2	86.67	80	−6.67	78	4	70.36	81.76	11.4					
39	2	86.67	80	−6.67	79	4	83.33	80	−3.33					
40	2	86.67	80	−6.67	80	4	63.33	70	6.67					

**FIGURE 3:**
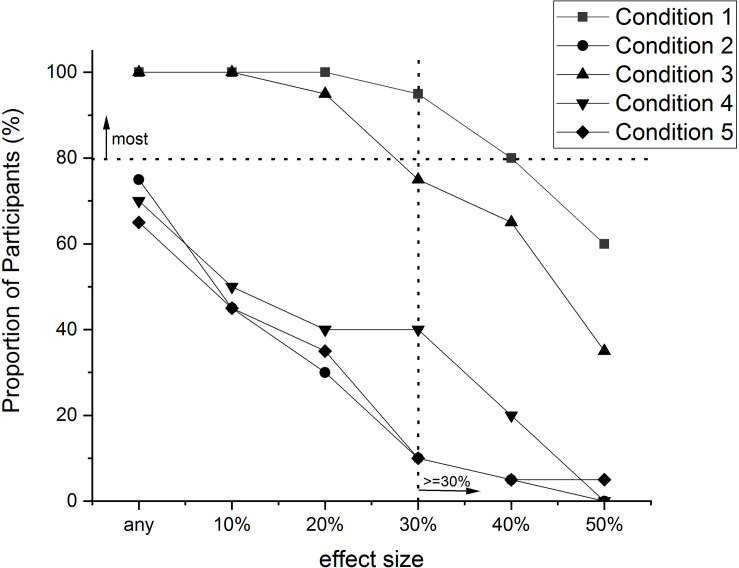
The proportion of people (%) in each condition of the Speelman et al. (2010) experiment that exhibited a particular “effect size” (i.e., a reduction in RT of a particular amount), or greater. Effect size was calculated as the difference between Pre-test RT and Post-test RT as a percentage of Pre-test RT [100 × (PreRT – PostRT)/PreRT]. Original data values are presented in Table 2.

**FIGURE 4:**
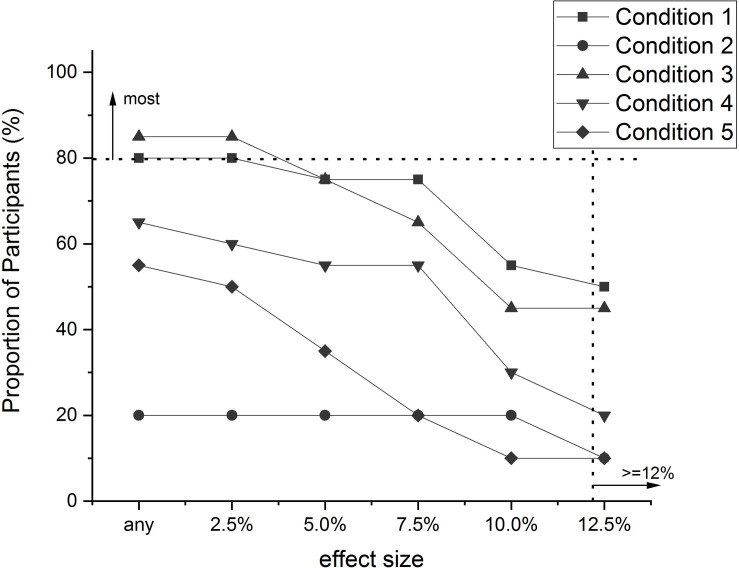
The proportion of people (%) in each condition of the Speelman et al. (2010) experiment that exhibited a particular “effect size” (i.e., an increase in Accuracy of a particular amount), or greater. Effect size was calculated as the difference between Post-test Acc and Pre-test Acc (PostAcc–PreAcc). Original data values are presented in Table 3.

In order to conduct a pervasiveness analysis such as we have outlined above, we first need to define the effect that we are interested in detecting. In the skin cancer experiment, the aim was to detect improvement differences between the conditions. In the original analysis, the size of the improvement was not central, just whether the mean improvement observed in some groups was greater or less than the improvement observed in other groups. However, an average amount by which the accuracy of detection improved was reported. In a pervasiveness analysis it is important to define the amount of improvement we think is important enough to warrant further consideration. This is the hard bit. We could take a relaxed view and consider that any improvement in RT would be sufficient to indicate an improvement. This would likely result in many people in each group being classed as having improved, particularly since for all participants in this design it would have been at least the second time they performed the task, and it could be expected that most would improve at least a small amount for just this reason. On the other hand, we could adopt a strict attitude, and only count reductions in RT that were, for example, 50% or greater as indicating improvement. This would likely reduce the number of people in each group exhibiting such an improvement by a large amount. To illustrate how the number of people showing an effect is related to the size of the effect we are interested in, the number of people in the skin cancer experiment who exhibited improvements in RT and Acc were counted, for improvements of different size. These counts are presented in Figures 3, 4.

The first thing to notice about both figures is the lines all decline in value as the effect sizes increase. This is to be expected, especially if the effects under observation have natural limits to their magnitude, as is the case here with RT and Accuracy.

In Figure 3, the differences between the conditions in their effects on improvements in RT are obvious. For any particular effect size there were more people in conditions 1 and 3 that exhibited this amount of improvement than people in the other conditions. Conditions 1 and 3 were the conditions in which participants practiced making decisions about skin lesions. Using the benchmark suggested above, we could conclude then that ‘most’ people (i.e., 80%) in these conditions improved their response speed by at least 30%. We have included in the figure a dotted horizontal line at the 80% mark on the proportion scale, and a dotted vertical line at the 30% mark on the effect size scale to assist with determining whether or not most people in a condition improved by this much or more. None of the other conditions reached this benchmark for any of the effect sizes. Therefore it seems safe to conclude from this data that, insofar as the sample was representative of the population, most people would improve the speed of their decision making in this task by at least 30% by engaging in the training utilized in conditions 1 and 3, whereas few people would improve by this amount by being subject to any of the other conditions.

The differences between the conditions with respect to the Accuracy of the decisions (Figure 4) are not as stark as for RT. Although more people in conditions 1 and 3 exhibited particular improvement amounts than people in the other conditions, as far as meeting the standard for ‘most’ people, the greatest amount of improvement exhibited by 80% of people in conditions 1 and 3 was only 2.5%. This is substantially smaller than the amount of improvement spruiked in the original report of this experiment (12–15%). This discrepancy does not reflect some sort of arithmetic error in the original report, just a difference in perspective. The discrepancy does, however, demonstrate how reliance on means can be misleading in terms of representing majority performance. Furthermore, this result has demonstrated how a pervasiveness analysis enables determination of whether a group effect reflects the performance of the majority of the group, or possibly the existence of a sub-set within the group that displayed a very large effect. Indeed, inspection of Table 3 indicates that there are some possible outliers in conditions 1 and 3 that would have had an inflationary effect on the means for each condition, which probably did not affect the outcome of the original analysis, but it certainly did affect the final conclusion about the amount of improvement observed. The pervasiveness analysis is not affected by such outliers to the same extent.

## Advantages of a Pervasiveness Analysis

An analysis that considers the pervasiveness of an effect within a sample has several advantages over an analysis that combines the data of members of a sample to determine whether there has been an overall effect. We consider some of these below:

### Superior Measure of Effect Size

A pervasiveness analysis will provide a superior measure of effect size to traditional measures of effect size. In the pervasiveness method, a (minimum) effect size is stated and can be plotted on a pervasiveness graph such as those in Figures 3, 4, and the proportion of people who meet this criterion is reported. In traditional measures, the effect size reported is an average calculated from the sample. For instance, the effect size stemming from an experiment that demonstrates a performance difference between two conditions is a reflection of the size of the difference between the mean performance in one condition with the mean performance in the other condition. Just as the mean of a set of scores does not convey how most of the sample behaved, traditional effect size measures do not reveal the size of the effect revealed by most people in the sample. In contrast, a pervasiveness analysis will indicate the size of the effect demonstrated by most people in the sample.

### Results Closer to What We Want to Say

Earlier we identified an issue with the way we interpret the results of experiments, where we regularly imply that our data indicates that *most* people behave in a certain way. The issue we identified was that we rarely look at our data in a way that will determine if these conclusions are a fair reflection of the behavior of most people in the sample we have studied. Conducting a pervasiveness analysis, however, provides clear evidence that can support such statements, when warranted, and bring our science a lot closer to the types of conclusions we seem to long for but which have been justified with insufficient evidence.

### Easily Combine Pervasiveness Estimates From Different Studies

The type of pervasiveness analysis outlined here results in values that are simple counts of the number of people in a sample who exhibit a particular type of performance. If other studies examine the same phenomenon with different samples, and report pervasiveness information, it would be a straightforward exercise to combine the various pervasiveness values into a new value that represents the total number of people exhibiting the target performance in the combined sample. For example, imagine five studies, with *N* of 40, 65, 90, 101, and 135. Imagine further that in each of these studies, the reported number of people who exhibited a particular effect was 35, 58, 82, 96, and 120, respectively. These results could be combined, in a form of meta-analysis, to indicate that the overall pervasiveness of the effect is 391/431, or over 90%. This result is arguably more convincing that the effect is demonstrated by most people than any of the results for the individual studies.

### Pervasiveness Is Similar to Replicability

A pervasiveness analysis not only provides information about how pervasive an effect is, it also can be interpreted as a measure of the replicability of the effect. That is, pervasiveness explicitly represents the proportion of people in a sample that show the effect. The pervasiveness of an effect also implies the probability of observing the effect when we test one more person, or another group of people. This suggests a means for increasing the replicability of results in Psychology: If the 80% rule is accepted, this could rule out small effects from being investigated further, and thus could reduce the publication of non-reproduceable effects.

### Low Pervasiveness May Indicate the Existence of Homogenous Sub-Groups

It is possible that, according to the 80% rule, no overall effect is observed in a sample. Closer scrutiny, however, of how individuals in the sample performed may indicate that there are sub-groups that behave in different ways. If there was some way of characterizing these groups (e.g., handedness), then it might be possible to generate more refined effect descriptions (e.g., 80% of left handers performed in *X* way, 80% of right handers performed in *Y* way). Such an analysis provides similar information to that often provided by individual differences analyses using a correlational approach, particularly those exploring the existence of moderating variables.

### The Benefits of Observing Effects Within Subjects

Given that a pervasiveness analysis as we have outlined here requires that each person in a sample can potentially exhibit an effect, it may not be possible to apply such an analysis with all experimental paradigms or designs. For instance, some within-subject designs are well-suited to analyses of pervasiveness. In such designs a measure of a change in performance can be compared with the pre-defined change that constitutes the effect. In contrast, between-subject comparisons could be challenging, especially if there is only one observation per person. In such cases, it may be necessary to reconceptualise the effect, or re-design the experiment, so that the effect can be observed within subjects. If this is not possible then an alternate method, such as OOM (Grice, 2011) could be useful. It is worthwhile noting, however, that between-subjects designs suffer a logical problem with respect to inferring experimental effects. For instance, the gold-standard of research design, the Randomized Control Trial (RCT) is always missing half of the necessary data to fully observe an effect (Eronen, 2020). That is, such experiments possess the implicit assumption that we can infer effects that occur within people by comparing people in one condition with people in another condition. In other words, between-subject effects can be used to infer within-subject effects. If we never observe these within-subject effects, how confident can we be that they exist? This uncertainty underlines the importance of trying to observe effects within subjects wherever possible, while recognizing the potential for sequence or carryover effects. The situation is made even more extreme when correlations are used to infer trend relationships between variables. In such studies, individuals contribute only one point in a trend. Indeed, “the between-person differences … simply do not exist at the level of the individual” (Lamiell, 2013, p. 70), further emphasizing the need to examine trends within individuals (Lamiell, 1997; Schmiedek et al., 2020).

The pervasiveness method of analysis does not preclude concern with measurement error. In many situations, it will not be sufficient to compare two measurements of a person to determine whether or not they have exhibited an effect because each of those two measurements may not provide an accurate indication of the person’s true value on the dependent variable of interest. If their performance on that variable is typically associated with a distribution of values, and reaction time is a classic example (Ratcliff, 1979), then it will be necessary to take many measurements within the two experimental conditions of interest. Such an approach may then require some form of inferential statistical test to determine whether the sample of measurements for the individual reflect the effect under scrutiny.

### Effects Have Clear *a priori* Definitions

One valuable initiative that has followed the reproducibility crisis in psychology has been the pre-registration of experiments. One advantage of pre-registering experiments prior to their onset is that many of the questionable research practices that have been identified as possible causes of poor reproducibility of psychological effects are rendered less influential (Nosek and Lakens, 2014; although see van Rooij, 2019). A similar advantage applies to pervasiveness analyses. These require a clear definition of what type of behavior constitutes an effect prior to performing the analysis. This could be derived from previous research, theory, or some other form of reasoning about what would constitute a scientifically significant effect. For example, a researcher could argue that an experimental condition that leads to 25% or more improvement in performance speed on a particular task compared to a control condition was an effect worth testing for. Decisions about what constitutes an effect would be intimately connected with the nature of the effect, and the possible causes underlying it. In contrast, sample-based statistical analyses often use the data to define an effect (e.g., the mean performance in condition A was faster than the mean performance in condition B), and provide a *post hoc* characterization of its magnitude (i.e., the effect size). Subsequent experiments on the same phenomenon are rarely concerned with finding the same sized effect, just with whether or not the effect, of any size, can be found at all. Requiring a clear *a priori* definition of an effect reduces the chances that an effect is defined by the data that is collected and so could be as much a product of the desire to obtain a statistically significant effect as a reflection of a real effect.

## Conclusion

The ergodic fallacy goes a long way to explain the reproducibility issue in psychology. If sample-level effects in our experiments are not reflections of something that exists in all or even most individuals in our samples, but are more a reflection of the idiosyncratic combination of idiosyncratic behaviors of the individuals, then any attempt to reproduce a set of effects with another sample of people will be doing so with a different combination of idiosyncratic behaviors (cf. Tang and Braver, 2020). It is no surprise at all, then, that many effects are difficult to replicate.

Given the concerns raised above, when should a researcher be worried about the methods they use in their own research? Consider the following questions:

(1) Does the design of an experiment assume that the behavior of each participant is an instantiation of the same basic psychological phenomenon (e.g., they have the same cognitive mechanisms, or they are subject to a common effect)?
(2) Is this assumption reinforced by the methods used in that they mask the possibility of the alternative?

If the answer to these questions is ‘yes,’ then a researcher should be worried. They are assuming their sample constitutes an ergodic system when this is unlikely to be justified *a priori*, and so, according to the ergodicity theorem, their research conclusions are not valid. That is, they are not justified in concluding that any effects they have observed at the sample level apply to any individuals, either in their sample, or in the population. The conclusions they draw about their research should be limited to the sample-level behaviors of other samples because they have observed phenomena that result from combining the behaviors of several people. Whether the phenomena are analogous to the behaviors of individuals remains an open question.

We have presented a relatively simple method for determining the pervasiveness of psychological effects. Although we consider the method can be straightforwardly applied to data collected in typical psychology experiments, and it provides a number of advantages over NHST techniques typically applied to those experiments, we expect great resistance to change from the majority of psychology researchers. Certainly previous attempts to change analysis behaviors have not been met with success (Speelman and McGann, 2013). We suggest two reasons for why this might be the case, one at the level of individual researchers, the other at the level of professional norms.

The first is a result of the tendencies of individual researchers. Having trained within a particular mode of defining and operationalising research questions, in accordance with the standard set of analytical tools available to us, shifting away from ergodic thinking would require a re-education which could look, on the face of it, to be rather daunting. If the rich value of such new analyses has not been fully grasped through their technical presentation, then the investment of precious time needed to get to grips with them will remain unlikely.

A second reason we believe underlies the inertia against responding to the ergodic problem is one that has been described by historians of psychological research for some time, and that is our granting of a professional imprimatur to a particular “standard” set of methods and tools which have come to define psychological research (Danziger, 1994; Stam, 2004; Green, 2015; Flis and van Eck, 2018). Over time professional practices within the science have substantially coalesced around a core set of themes and processes, into which a very diverse set of research questions and topics have been fit. Professional incentives have thus all been structured according to the principles of ergodic thinking. Even the recent, dramatic discussions around our professional incentives and methodological practices have remained focused on how to do such ergodic-thinking research well, and the question, now with decades-long arguments of methodologists behind it, of whether we should be doing it at all has barely been raised.

These different kinds of reasons underlying our professional inertia in this regard warrant different kinds of responses, a two-pronged approach to tackling problems related to the non-ergodicity of human beings, which we might think of as *fireproofing*, and *firefighting*. Fireproofing involves substantive institutional and structural changes to the practices of professional psychological research. Such changes will mean changes to education and training, significant revision of our research methods and procedures, as well as shifts in standards and norms around reviewing and publishing of research. These kinds of systemic changes are difficult, costly, and can only be managed through large coordinated efforts by many people - it’s more like making a tide than making waves, more like building houses to be more fire-proof in the first place, than trying to put out fires when they happen.

Fireproofing is long-term thinking that may not show immediate or striking benefits. There are, however, some positive actions which do offer such immediate reward, and which can be managed without the investment of effort needed for something like training in an entirely new set of statistical techniques. Fire-fighting the ergodic fallacy can be done in a manner that does not undermine the worth of psychological research to date, but in fact means that many of the datasets we have already available contain rich veins of un-tapped value that we can access easily, and without major investment of time or resources.

An immediate first step, however, is one that can be undertaken without tearing at the foundations of what we do. In this paper we offer the straightforward consideration of the pervasiveness of effects as a fire-fighting measure, a means to explore our existing data in a manner that offers key insights to questions in which we are interested. In time, and with sufficient researcher interest, this could lead to a greater focus on observing the behavior of individuals with a view to discovering not only how they behave in particular circumstances, but the extent to which they all behave the same way. Addressing this problem extensively requires systemic change in our professional practices, a re-fitting of our methods and analytical techniques from the ground up in the service of the kinds of questions that psychologists are actually interested in answering. Such fireproofing of future research work is no small task, though the various movements for improved theorizing and research extant in the discipline at present suggests that such an overhaul is at least possible. As individual researchers we might begin to explore such new methods and consider revising our approach.

## Author Contributions

All authors listed have made a substantial, direct and intellectual contribution to the work, and approved it for publication.

## Conflict of Interest

The authors declare that the research was conducted in the absence of any commercial or financial relationships that could be construed as a potential conflict of interest.
